# Household transmission of human metapneumovirus and seasonal coronavirus

**DOI:** 10.1017/S0950268824000517

**Published:** 2024-05-21

**Authors:** Cristalyne Bell, Cecilia He, Derek Norton, Maureen Goss, Guanhua Chen, Jonathan Temte

**Affiliations:** 1 Department of Family Medicine and Community Health, School of Medicine and Public Health, University of Wisconsin, Madison, WI, USA; 2 Department of Biostatistics and Medical Informatics, School of Medicine and Public Health, University of Wisconsin, Madison, WI, USA

**Keywords:** human metapneumovirus, seasonal coronavirus, acute respiratory infections, coronavirus OC43, coronavirus HKU1

## Abstract

We analyzed data from a community-based acute respiratory illness study involving K-12 students and their families in southcentral Wisconsin and assessed household transmission of two common seasonal respiratory viruses – human metapneumovirus (HMPV) and human coronaviruses OC43 and HKU1 (HCOV). We found secondary infection rates of 12.2% (95% CI: 8.1%–17.4%) and 19.2% (95% CI: 13.8%–25.7%) for HMPV and HCOV, respectively. We performed individual- and family-level regression models and found that HMPV transmission was positively associated age of the index case (individual model: *p* = .016; family model: *p* = .004) and HCOV transmission was positively associated with household density (family model: *p* = .048). We also found that the age of the non-index case was negatively associated with transmission of both HMPV (individual model: *p* = .049) and HCOV (individual model: *p* = .041), but we attributed this to selection bias from the original study design. Understanding household transmission of common respiratory viruses like HMPV and HCOV may help to broaden our understanding of the overall disease burden and establish methods to prevent the spread of disease from low- to high-risk populations.

## Key results

- Infection of school-aged children is an important driver of respiratory virus transmission within households.
- As the age of an index case increases, the likelihood of human metapneumovirus transmission increases within households.
- As household density increases, the likelihood of seasonal coronavirus transmission increases within households.

## Introduction

Human metapneumovirus (HMPV) and human coronavirus (HCOV) are RNA viruses that commonly cause seasonal acute respiratory illness (ARI). In temperate climates, HMPV and HCOV often circulate in winter and early spring and can infect individuals of all ages multiple times over the course of their lives [1–3]. The viruses primarily cause respiratory symptoms that are usually mild to moderate in healthy adolescents and adults, but can lead to severe illness in young children, the elderly, and individuals with compromised immune systems [4–10]. Since people are unlikely to seek medical attention for a mild to moderate illness and laboratory testing can be limited in clinical settings, it is difficult to assess the magnitude of outbreaks and overall disease burden. There are currently no approved treatments beyond supportive care, making prevention a crucial part of protecting at-risk populations [11].

Most of what is known about HMPV and HCOV comes from clinical research focused on individual infections among patients [12]. We assessed household transmission rates for HMPV and HCOV using data collected for a community-based respiratory virus study where over 80% of participating families indicated they did not intend to seek medical care [13].

## Methods

The ORegon CHild Absenteeism due to Respiratory Disease Study (ORCHARDS) is a community-based prospective study in the Oregon School District, located in southcentral Wisconsin. Students within the school district are eligible to participate if they have (1) at least two acute respiratory symptoms (rhinorrhea, nasal congestion, sneezing, sore throat, cough, fever) that began within 7 days of contacting research staff and (2) a Jackson Score of 2 or more [14–16]. The Jackson score is a symptom-scoring system commonly used to assess viral illnesses. It is calculated by summing the severity points (0 = absent, 1 = mild, 2 = moderate, 3 = severe) for eight common cold symptoms (nasal discharge, nasal obstruction, sneezing, sore throat, cough, malaise, chilliness, and headache).

ORCHARDS began recruiting K-12 students on 5 January 2015. The following year, starting 6 January 2016, student families could participate in an optional supplementary study examining household transmission of influenza. Families could re-enrol in ORCHARDS and the supplementary household study if new symptoms began 30 days or 7 days after previous participation, depending on whether their illness occurred outside of an influenza season or during an influenza season. Each time a family participated, they were considered a unique episode and could be counted multiple times throughout the study period.

When students enrolled in ORCHARDS, research staff visited their homes to collect student demographic and illness information, a nasal swab for rapid influenza diagnostic testing, and either a nasopharyngeal (NP) or an oropharyngeal (OP) swab for reverse transcriptase polymerase chain reaction (RT-PCR). Families who consented to the household transmission study received supplies for participating family members to collect their own nasal swabs on the day of the initial ORCHARDS visit and an additional swab 7 days later. Research staff retrieved Day 0 and Day 7 specimens from all family members (including a self-collected Day 7 swab from the ORCHARDS student) and shipped them via courier to the Wisconsin State Laboratory of Hygiene for molecular testing.

### Diagnostics

All specimens from ORCHARDS students and household members were tested for influenza A and B viruses and Human Ribonuclease P using the in vitro diagnostic FDA-approved CDC Human Influenza Virus Real-time RT-PCR Diagnostic Panel (Cat.# FluiVD03) [17]. Day 0 staff-collected specimens from students were also tested for HMPV, HCOV, and other respiratory viruses using the multiplexed RT-PCR respiratory pathogen panel (Luminex NxTAG Respiratory Pathogen Panel) [18]. Aliquots of all residual specimens were archived at ≤−70 °C at the Wisconsin State Laboratory of Hygiene. Additional information on original study design can be found elsewhere [13, 19].

For this analysis, we performed retrospective testing on archived specimens collected from (1) symptomatic and asymptomatic family members if the ORCHARDS student was positive for HMPV, and (2) symptomatic family members if the student was positive for coronavirus OC43 or HKU1. Coronavirus OC43 and HKU1 are beta coronaviruses, which are also responsible for severe acute respiratory syndrome (SARS-2002), Middle Eastern respiratory syndrome (MERS-2012), and coronavirus disease (COVID/SARS-2019). We did not distinguish between HMPV strains.

Index cases for each household were determined by the first date of test positivity, or the first date of reported symptom onset if the date of tests positivity was tied. In one family instance for HMPV, a tie still existed, and both members were considered as index, using the worst symptom severity between the two, and index symptoms reported as occurring between either index.

### Statistical analysis

We performed individual and family-level regression models to assess factors that may be associated with HMPV and HCOV household transmission. The family-level model was a binomial regression with the outcome being the proportion of the family who tested positive for HMPV or HCOV (excluding the index case) within 7 days of the initial ORCHARDS visit. Covariates were the age of the index case, household density (number of family members divided by number of bedrooms in the household), and severity of the index case symptoms based on a scale of 1 (mild), 2 (moderate), or 3 (severe). Family size (number of non-index consenting family members) was accounted for in the model as a weight. For the individual-level model, a mixed effects binomial regression model was used, with each of the non-index case member’s HMPV or HCOV infection status (within 7 days) as the outcome, and with covariates of index case age, index case symptom severity, household density, and the age of the individual family member for the outcome observed. The family cluster was incorporated as a random intercept in the model.

Secondary analyses examined specific symptoms of the index case that were present, instead of index case severity. The model structures were the same as described above for both the family and individual-level models for both viruses, except one of the five following symptoms replaced index severity: fever, cough, rhinorrhea, malaise, and nasal congestion. Due to multicollinearity concerns, severity was removed when individual symptoms were examined. For each model type and virus type (individual- and family-level; HMPV and HCOV), the *p*-values for the set of five symptoms were also subjected to a Benjamini–Hochberg correction controlling false discovery rate at 5% before statistical significance was determined. Statistical significance was assessed at the 5% level. All analyses were performed in *R version 4.1.0* [20], with the *lme4* package [21] used for fitting mixed effects models and *ggplot2* [22] for graphics.

### Project approval

All components of ORCHARDS were reviewed and approved by the Human Subjects Committees of the Education and Social/Behavioral Sciences Internal Review Board (IRB) and the University of Wisconsin Health Sciences IRB (initial approval on 4 September 2013; ID number: 2013–1268) and the University of Wisconsin Health Sciences-IRB (initial approval on 5 December 2013, with additional approvals as the protocol expanded and modified; ID number: 2013–1357). The study is in full compliance with the Health Insurance Portability and Accountability Act of 1996 (HIPAA), the Family Educational Rights and Privacy Act (FERPA), and all other federally mandated human subjects.

## Results

Between 5 January 2015 and 12 March 2020, there were 1394 families who consented to the ORCHARDS household transmission study. We performed retrospective testing on samples collected from 128 families, where the Day 0 staff-collected sample from the ORCHARDS student was either positive for HMPV or HCOV (Table 1).

**Table 1.** Participant characteristics and illness presentation resulting from human metapneumovirus (HMPV) and human coronaviruses OC43 and HKU1 (HCOV)

Characteristics	HMPV	HCOV
Total participants	285	239
Mean age (SD)	23.46 (16.64)	23.35 (16.94)
<18 years old, *n*(%)	150 (52.6)	133 (55.6)
Female, *n* (%)	143 (50.2)	107 (44.8)
Family role, *n* (%)		
Student	71 (24.9)	57 (23.8)
Grandparent	0 (0)	2 (0.8)
Other adult	0 (0)	1 (0.4)
Parent	133 (46.7)	102 (42.7)
Sibling	81 (28.4)	77 (32.2)
Mean household density (SD)	0.91 (0.29)	1.05 (0.32)
Virus detected, *n* (%)	98 (34.4)	89 (37.2)
Secondary infection risk, % (95% CI)	12.2% (8.1–17.4)	19.2% (13.8–25.7)
Days from symptom onset, mean (range)	1.09 (0–11)	1.58 (0–10)
Index cases, *n* (%)	72^a^ (25.3)	57 (23.8)
Index symptoms^a^		
Rhinorrhea, *n* (%)	61 (85.9)	50 (87.7)
Nasal congestion, *n* (%)	68 (95.8)	49 (86.0)
Sore throat, *n* (%)	53 (74.6)	34 (59.6)
Cough, *n* (%)	68 (95.8)	47 (82.5)
Malaise, *n* (%)	60 (84.5)	41 (71.9)
Chills, *n* (%)	44 (62.0)	23 (40.4)
Headache, *n* (%)	41 (57.7)	29 (50.9)
Fever, *n* (%)	47 (66.2)	25 (43.9)
Index illness severity, *n* (%)		
None	0 (0)	1 (1.8)
Mild	8 (11.3)	18 (31.6)
Moderate	58 (81.7)	35 (61.4)
Severe	5 (7.0)	3 (5.3)

a In one of the 71 HMPV families in the analyses, a tie for index case was determined (same reported positive test dates and symptom start dates), and both members were considered as index. Specific symptoms were considered as occurring in either subject, and the worst illness severity between the two was used. Thus the *N* for specific symptoms and severity for the index in the HMPV analyses is out of 71, not 72.

### Human metapneumovirus

Seventy-one families comprised of 285 individuals were considered for this analysis because the ORCHARDS student tested positive for HMPV. There were 20 families with possible household transmission where at least one secondary infection of HMPV was detected. There were 72 index cases and 213 non-index cases. The index case was determined by the date of symptoms onset. For one family, we were unable to determine the index case because two family members had the same onset date.

The secondary infection rate for households was 12.2% (95% CI: 8.1%–17.4%) with 26 out of 213 non-index family members contracting HMPV. The average time for transmission from an index to a secondary case (difference between symptom onset for HMPV-positive members) was 5.2 days (±3.26). The transmission assessment period was 14 days (7 days before the initial household visit and the 7 days between Day 0 and Day 7 sample collection). The minimum documented transmission time was 0 day and the maximum time was 11 days. We detected one case of asymptomatic infection of HMPV among a non-index family member.

**Figure 1.**
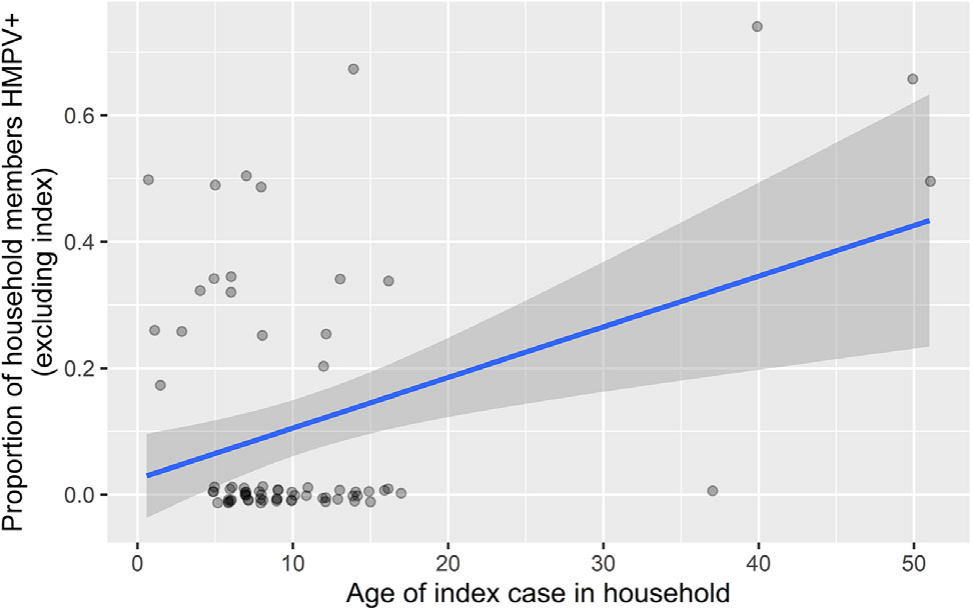
Age of index case and proportion of other household members having human metapneumovirus.

Age of index case was positively associated with testing positive for HMPV in both the individual model (OR = 1.05, *p* = 0.004) and family model (OR = 1.06, *p* = 0.016; (Table 2 and Figure 1). In the individual model, age of the non-index case was negatively associated with the likelihood of testing positive for HMPV during the 7-day monitoring period (OR = 0.97, *p* = 0.049). Index case severity and sleeping density were not statistically significant in either model. When we substituted individual symptoms for symptom severity, none of the five symptoms of interest (fever, cough, rhinorrhea, malaise, and nasal congestion) were significant for HMPV in either the family or individual-level models.

**Table 2.** Human metapneumovirus household transmission regression

Model	Covariate	OR	OR CI	*p*-value
Family	Household density	2.21	0.60–8.2	0.234
	Index case severity	1.66	0.56–4.98	0.363
	Index case age (years)	1.05	1.02–1.09	0.004*
Individuals	Household density	1.91	0.31–12.01	0.488
	Index case severity	1.82	0.45–7.33	0.399
	Index case age (years)	1.06	1.01–1.10	0.016*
	At–risk / non–index subject age (years)	0.97	0.94–1.00	0.049*

* *p*-value ≤ 0.05.

### Human coronavirus OC43 and HKU1

Fifty-seven families comprised of 239 individuals were considered for this analysis because the ORCHARDS student tested positive for either HCOV OC43 or HKU1. Twenty-one families had a possible case of household transmission where at least one secondary infection was detected. The secondary infection rate for households was 19.2% (95% CI: 13.8%–25.7%) with 35 out of 182 non-index family members contracting HCOV. The average time for transmission from index to secondary case was 4.4 days (± 3.2). The minimum documented transmission time was 0 days and the maximum time was 10 days.

**Figure 2.**
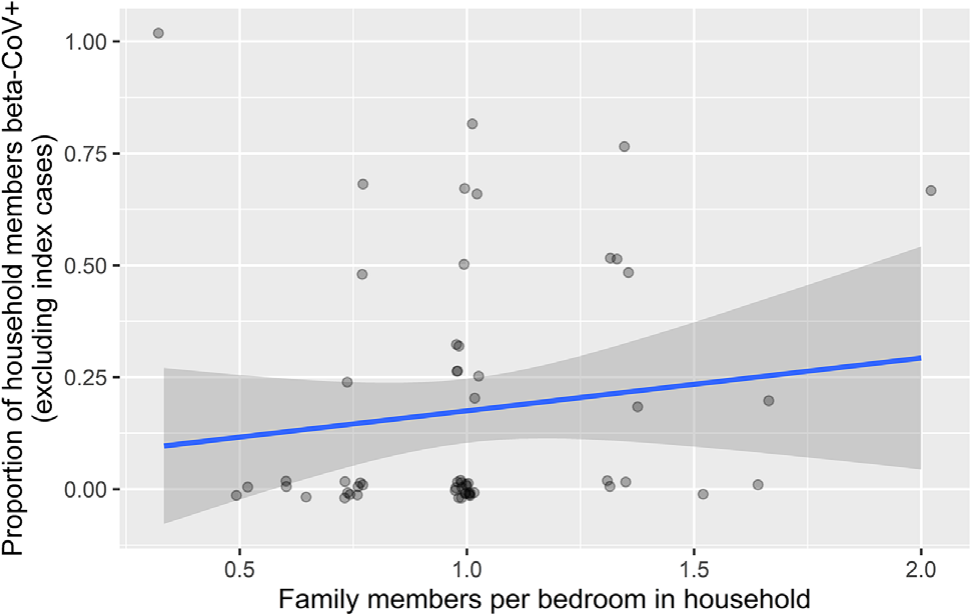
Household density and proportion of other family members having coronavirus.

In the family-level regression, number of family members per bedroom was positively associated with the proportion of the family members who contracted the virus (OR = 4.81, *p* = 0.048; Table 3 and Figure 2). In the individual model, age of the non-index case was negatively associated with the likelihood of testing positive for HCOV (OR = 0.97, *p* = 0.041; Table 3). Index case severity and index case age were not statistically significant in either model, nor were individual symptoms (fever, cough, rhinorrhea, malaise, and nasal congestion) when substituted them for severity.

**Table 3.** Human coronaviruses OC43 and HKU1 household transmission regression

Model	Covariate	OR	OR CI	*p*-value
Family	Household density	4.81	1.01–22.8	0.048*
	Index case severity	1.32	0.56–3.10	0.525
	Index case age (years)	1.02	0.97–1.07	0.431
Individuals	Household density	3.68	0.55–24.47	0.178
	Index case severity	1.02	0.38–2.72	0.968
	Index case age (years)	1.01	0.96–1.07	0.681
	At–risk/non–index subject age (years)	0.97	0.95–1.00	0.041*

* *p*-value ≤ 0.05.

## Discussion

We assessed household transmission of HMPV and HCOV (OC43 and HKU1) and found a secondary infection rate of 12.2% and 19.2%, respectively. Using individual- and family-level regression models, we also examined possible factors that could have contributed to household transmission (age, severity of illness, individual symptoms, and household density). In both models, the age of index case was positively associated with individuals testing positive for HMPV. A positive association between household density and the proportion of family members who contracted HCOV was detected in the family-level model. The only statistically significant finding that HMPV and HCOV had in common was a negative association between the age of non-index case and the likelihood of testing positive. This association is likely the result of recruitment bias since family participation was dependent on a K-12 student being sick.

### Secondary infections risk

There is limited research available on secondary infection or transmission rates for HMPV in the literature for comparison. In a case report on the unexpected death of a 33-month-old infected with HMPV, health authorities found a prevalence of 36% among the 22 tested children who attended the same daycare as the child who died [12]. One study in Japan looked at HMPV infection among family members, but researchers were unable to determine the total frequency of HMPV infections within households because they were limited to testing family members who presented to paediatric outpatient clinics [23]. For the family members who visited the clinics, the average time between cases was 4–5 days, which was comparable to our findings. In the broader literature, the average timeframe is thought to be between 4 and 9 days [24].

Following the COVID-19 pandemic, household studies assessing transmissibility of seasonal coronavirus and SARS-coronavirus-2 (SARS-CoV-2) have become more common. Some studies reported a secondary infection rate over 50% for SARS-CoV-2 [25]. In contrast, seasonal coronavirus tends to be lower. One study reported an overall secondary infection rate of 8% and a median symptom onset of 7 days [26]. Another study found a secondary infection risk that ranged from 7.2% to 12.6%, depending on the seasonal strain [27]. Our secondary infection rate is closer to some of the SARS-CoV-2 estimates, but that may be due to differences in study design.

Additionally, the methods used here assumes that non-index infections are from the index case themselves, and not from outside the family, or secondary cases in the family, which may upward bias the secondary infection estimates in this study versus reality.

### Factors contributing to household transmission

The positive association found in the family-level model between index case age and likelihood of spreading HMPV to other family members should be interpreted carefully. There were only four instances where a parental figure was the index case, and this cluster could be driving the association, though having a head-of-household as the index may represent greater infection risk for the family. If an index indicator of child versus adult is used instead of index age, significant positive association is maintained between an adult index and the risk of secondary infections. However, a positive association was also detected in the individual model, where the presence of potential bias is at play. Since students are represented in the lower age cluster, we would expect some of the proportion values to be lower than they are, given that student respiratory symptoms are a pre-requisite for study inclusion. Thus, the association between index case age and likelihood of spreading HMPV is potentially stronger than what we observed. Our findings are also supported by the literature. Some studies have indicated that children younger than 5 years are the most susceptible to HMPV infection, and in some cases, younger siblings are likely to acquire HMPV infection from their older siblings [23, 28].

Average household density was slightly higher among the HCOV group than the HMPV group, which might have contributed to a higher secondary infection rate. The relatively small sample size may explain why the positive association between household density and the proportion of family members contracting HCOV only showed up in the family-level model and was not seen in the HMPV models. The definition of household density is also imprecise because it is based on the number of bedrooms in a household and does not account for instances where family members share bedrooms.

### Limitations

There were additional limitations in this study. Notably, if the student was not the index case in the house, the student would still be positive for HMPV or HCOV since family member specimens would not have been tested for either virus if the student had not initially tested positive. Instances where a non-student family member fails to pass HMPV or HCOV on to a student are likely underreported because the family would not meet the initial inclusion criteria of having a student in the household with respiratory symptoms. It is also possible that the true index case recovered before enrolling in the study, or an additional family member became ill after the observational period. We may catch some infections prior to the student becoming ill and qualifying for the study by asking if anyone had symptoms 7 days prior to the ORCHARDS visit, but we are unable to determine the extent of spread beyond the enrollment period. Thus, findings such as the average time for transmission may be limited due to the 14-day transmission assessment period. Alternatively, it is also possible we missed infections from days 1–6, if the individual became ill and recovered between sample collection days. However, this is less likely given the characteristics of both viruses [1, 4] and sensitivity of the RT-PCR test [18].

## Conclusion

Most data on seasonal respiratory viruses are collected in clinic and hospital settings, which provides little insight into transmission within families and the broader community. This approach to disease surveillance can be more convenient and cost-effective, but it limits our understanding of the overall disease burden. Community studies provide a broader picture of transmission patterns, which can help inform preventative measures designed to keep people out of clinics and hospitals. HMPV and HCOV are two of the many respiratory viruses that can be acquired at school and easily spread to other family members. Age and household density may play a role in how many family members are likely to contract the virus. More research is needed to understand whether the same can be said for other common respiratory viruses.

## Data Availability

Data can be found through Harvard Dataverse: https://doi.org/10.7910/DVN/HVOZI8.
